# Selenium Nanoparticles as Candidates for Antibacterial Substitutes and Supplements against Multidrug-Resistant Bacteria

**DOI:** 10.3390/biom11071028

**Published:** 2021-07-14

**Authors:** Hee-Won Han, Kapil D. Patel, Jin-Hwan Kwak, Soo-Kyung Jun, Tae-Su Jang, Sung-Hoon Lee, Jonathan Campbell Knowles, Hae-Won Kim, Hae-Hyoung Lee, Jung-Hwan Lee

**Affiliations:** 1 Institute of Tissue Regeneration Engineering (ITREN), Dankook University, Cheonan 31116, Korea; 12201075@dankook.ac.kr (H.-W.H.); dynamic2020@korea.ac.kr (K.D.P.); j.knowles@ucl.ac.uk (J.C.K.); kimhw@dku.edu (H.-W.K.); 2 Department of Biomaterials Science, College of Dentistry, Dankook University, Cheonan 31116, Korea; 3 Department of Materials Science and Engineering, Korea University, Seoul 02841, Korea; 4 Department of Life Science, Handong Global University, Pohang 37554, Korea; jhkwak@handong.edu; 5 Department of Dental Hygiene, Hanseo University, Seosan 31962, Korea; iris979@hanseo.ac.kr; 6 Department of Pre-Medi, College of Medicine, Dankook University, Cheonan 31116, Korea; jangts@dankook.ac.kr; 7 Department of Oral Microbiology and Immunology, College of Dentistry, Dankook University, Cheonan 31116, Korea; dennisyi@dankook.ac.kr; 8 UCL Eastman-Korea Dental Medicine Innovation Centre, Dankook University, Cheonan 31116, Korea; 9 Division of Biomaterials and Tissue Engineering, Eastman Dental Institute, University College London, London NW3 2PF, UK; 10 Department of Nanobiomedical Science and BK21 PLUS NBM Global Research Center for Regenerative Medicine, Dankook University, Cheonan 31116, Korea; 11 Cell & Matter Institute, Dankook University, Cheonan 31116, Korea; 12 Department of Regenerative Dental Medicine, College of Dentistry, Dankook University, Cheonan 31116, Korea

**Keywords:** multidrug-resistant bacteria, antibacterial activity, selenium nanoparticles, synergistic effect

## Abstract

In recent years, multidrug-resistant (MDR) bacteria have increased rapidly, representing a major threat to human health. This problem has created an urgent need to identify alternatives for the treatment of MDR bacteria. The aim of this study was to identify the antibacterial activity of selenium nanoparticles (SeNPs) and selenium nanowires (SeNWs) against MDR bacteria and assess the potential synergistic effects when combined with a conventional antibiotic (linezolid). SeNPs and SeNWs were characterized by transmission electron microscopy (TEM), X-ray diffraction (XRD), Fourier transform infrared spectroscopy (FTIR), zeta potential, and UV-visible analysis. The antibacterial effects of SeNPs and SeNWs were confirmed by the macro-dilution minimum inhibitory concentration (MIC) test. SeNPs showed MIC values against methicillin-sensitive *S. aureus* (MSSA), methicillin-resistant *S. aureus* (MRSA), vancomycin-resistant *S. aureus* (VRSA), and vancomycin-resistant enterococci (VRE) at concentrations of 20, 80, 320, and >320 μg/mL, respectively. On the other hand, SeNWs showed a MIC value of >320 μg/mL against all tested bacteria. Therefore, MSSA, MRSA, and VRSA were selected for the bacteria to be tested, and SeNPs were selected as the antimicrobial agent for the following experiments. In the time-kill assay, SeNPs at a concentration of 4X MIC (80 and 320 μg/mL) showed bactericidal effects against MSSA and MRSA, respectively. At a concentration of 2X MIC (40 and 160 μg/mL), SeNPs showed bacteriostatic effects against MSSA and bactericidal effects against MRSA, respectively. In the synergy test, SeNPs showed a synergistic effect with linezolid (LZD) through protein degradation against MSSA and MRSA. In conclusion, these results suggest that SeNPs can be candidates for antibacterial substitutes and supplements against MDR bacteria for topical use, such as dressings. However, for use in clinical situations, additional experiments such as toxicity and synergistic mechanism tests of SeNPs are needed.

## 1. Introduction

Since the discovery of penicillin, many antibiotics have been developed. However, the number of bacteria that are resistant to antibiotics is increasing due to the misuse and abuse of antibiotics. Among these, when a bacterium is resistant to three or more antimicrobial classes, the bacterium is referred to as multidrug-resistant (MDR) [1]. MDR bacteria are a major problem that threatens human health [2,3]. MDR bacteria are the leading cause of nosocomial infections, and outcomes for patients infected with MDR bacteria tend to be worse than for patients infected with bacteria that are more sensitive to antibiotics [4,5,6]. Therefore, there is an urgent need to identify new substances that can supplement conventional antibiotics or target MDR bacteria.

Many experiments have been performed in various fields, such as tissue engineering, to determine the antibacterial effects of nonantibiotic materials [7,8,9]. One of the nonantibiotic materials with antibacterial activity is a natural product. The antimicrobial activity of natural products has been reported in many papers, and some of these products even show antimicrobial effects against MDR bacteria [10,11,12,13,14,15]. Another candidate for nonantibiotic materials is antibacterial nanoparticles. Recently, antibacterial experiments using nanoparticles have been actively conducted. Selenium, silver, gold, and ZnO nanoparticles also showed antibacterial activity against MDR bacteria as well as strains that are not resistant to antibiotics [16,17,18,19,20,21,22,23,24,25].

Nanoparticles are attracting attention given their very small size and various antibacterial properties. Since nanoparticles are very small, there are many opportunities to interact with bacteria per unit area, which can make the antibacterial activity of nanoparticles more powerful. Nanoparticles can also initiate several bactericidal pathways, such as disrupting the bacterial membrane and release of intracellular components, making it difficult for bacteria to become resistant [26,27]. Among them, selenium nanoparticles (SeNPs) have attracted increased attention. Selenium (Se) is an essential trace element for humans (about 50 µg/day), and antimicrobial studies using different forms of Se, such as selenite and SeNPs, have been reported [28,29,30,31,32,33]. Se is a significant element, because it is an important cofactor for antioxidant enzymes such as glutathione peroxidase and thioredoxin reductase [34,35]. One study found that selenium deficiency can damage the liver, heart, kidneys, skeletal muscle, and testes [36]. However, doses above 400 µg/day can be toxic, and studies on the side effects of Se treatment such as diabetes and prostate cancer have been reported [32,37,38,39]. Se toxicity may arise through some mechanisms, such as oxidative stress or substitution of selenium for sulfur during protein assembly [40,41]. However, these Se toxicities depend on the forms of Se. For example, selenium nanoparticles (SeNPs) showed better bioavailability and less toxicity compared with inorganic and organic Se forms, such as sodium selenite (inorganic form of Se) and selenomethionine (organic form of Se) [42,43,44]. Therefore, SeNPs are getting more attention than other Se forms, and we also focused on SeNPs in this study.

In this study, the antibacterial activities of SeNPs and selenium nanowires (SeNWs) were analyzed by macro-dilution minimum inhibitory concentration (MIC) tests against methicillin-sensitive *Staphylococcus aureus* (MSSA), methicillin-resistant *S.* *aureus* (MRSA), vancomycin-resistant *S. aureus* (VRSA), and vancomycin-resistant enterococci (VRE). Based on the results from this macro-dilution MIC test, only SeNPs showed antibacterial effects against MSSA, MRSA, and VRSA. Therefore, the purpose of this study was to evaluate the antibacterial activity of SeNPs against MDR bacteria, identify the synergistic effects of SeNPs with linezolid (LZD), and determine the mechanism of synergism.

## 2. Materials and Methods

### 2.1. Materials

Sodium selenite (Na_2_SeO_3_; Sigma-Aldrich, St. Louis, MO, USA), L-ascorbic acid (C_6_H_8_O_6_, Sigma-Aldrich, USA), D-(+) glucose (C_6_H_12_O_6_, Sigma-Aldrich, St. Louis, MO, USA), sodium hydroxide beads (NaOH, Daejung, Seohaean-ro, Siheung, Korea), and 1 N hydrochloric acid (HCL, Daejung, Seohaean-ro, Siheung, Korea) were used in these studies. All chemicals were reagent grade and used without further purification.

### 2.2. Synthesis of SeNPs and Nanowires

SeNPs and SeNWs were selectively synthesized by reducing aqueous solutions of sodium selenite with ascorbic acid and glucose respectively, as previously reported [45,46]. In a typical procedure of synthesizing SeNPs, 100 mM of sodium selenite aqueous solution was mixed with 100 mM of ascorbic acid, and the solution pH was adjusted to 7.1 using NaOH or HCl. The resulting solution color changed to red in 5–8 min, and the reaction was continued for the next 3 h and the solution was centrifuged at 15,000× *g* for 30 min. Then, the sample was obtained in suspended pellet (not fully pellet) form and washed with distilled water (DW). The final SeNPs-DW suspension solution was lyophilized to collect the selenium nanoparticles. SeNWs were synthesized by the addition of 2 g of glucose in 320 mL of aqueous solution of 100 mM sodium selenite in a 500 mL glass bottle and mixed for 30 min with vigorous magnetic stirring. The mixture solution was tightly sealed in the bottle. The bottle was placed in an electrically controlled oven and maintained at a constant temperature of 90 °C for 12 h. The turbid brick-red color solution was then quenched in 300 mL of cold water, and the product was collected by centrifugation at 15,000× *g* for 5 min. The sample was washed thrice with DW to remove impurities. The brick-red color product was redispersed in 200 mL of absolute ethanol in a 300 mL glass bottle. The bottle was sealed, covered with aluminum foil, and placed in darkness at room temperature for further growth of SeNWs. After a week, a sponge-like blackish-red jelly precipitate formed at the bottom of the bottle, and the upper part of the solution became colorless. Finally, SeNWs were collected by spongy precipitate centrifugation, washed with absolute ethanol and DW, and dried using a freeze dryer (TFD5503, Ilshin Lab. Co. Ltd., Eunhyeon-myeon, Yangju, Korea) for further use.

### 2.3. Characterizations of SeNPs and SeNWs

The morphology and size, crystalline phase, chemical functional groups, and surface charge of SeNPs and SeNWs were characterized by transmission electron microscopy (TEM; JEOL-7100), X-ray diffraction (XRD; Rigaku, Tokyo, Japan), Fourier transform infrared spectroscopy (FTIR; 640-IR, Varian, VIC, Australia), zeta potentials (Zetasizer Nano; Malvern Instrument, Malvern, UK), and UV-visible analysis (UV-Vis; Varian Cary 100, Agilent Technologies, Paolo Alto, CA, USA).

### 2.4. LC-MS/MS Analysis for N-Formylmethionyl-Leucyl-Phenylalanine (fMLP)

Liquid chromatography/tandem mass spectrometry (LC-MS/MS) was performed with an Ultimate 3000 RS-Q-Exactive Orbitrap Plus (Thermo Fisher Scientific, Waltham, MA, USA) at the Yonsei Center for Research Facilities (YCRF, Seodaemun-gu, Seoul, Korea). For the negative-mode LC condition, Acquity UPLC BEH C18 (1.7 µm, 2.1 × 100 mm) was used as an LC column at 50 °C. The injection volume was 10 µL. The run time was 17 min. The mobile phase consisting of solvent A, 6.5 mM ammonium bicarbonate in DW, and solvent B, 6.5 mM ammonium bicarbonate in methanol (MeOH), was delivered at a flow rate of 0.4 mL/min. The following linear gradient was used: 0 min, 0% B; 1 min, 10% B; 14 min, 100% B; 17 min; 10% B. The electrospray ionization (ESI) (negative ionization mode) conditions were as follows: capillary voltage was 2.5 kV, S-lens RF level was 50, capillary temperature was 263 °C, and aux gas heater temperature was 425 °C. The sheath and aux gas flows were 50 and 13, respectively.

### 2.5. Bacterial Strains and Culture Conditions

*S. aureus* ATCC6538 (MSSA) and *S. aureus* MU50 ATCC700699 (MRSA) were purchased from American Type Culture Collection (ATCC, Manassas, VA, USA). VRSA48 and VRE (vancomycin-resistant *Enterococcus*) c6485, which were clinically isolated, were gently provided by the Korea Research Institute of Bioscience & Biotechnology (KRIBB, Yuseong-gu, Daejeon, Korea) and Seoul National University (SNU, Gwanak-gu, Seoul, Korea) respectively, through Prof. Jin-Hwan Kwak of Handong Global University (HGU, Buk-gu, Pohang, Korea). All strains that were kept as glycerol stock solutions in a −80 °C deep freezer were streaked on Mueller-Hinton agar (MHA, Difco Laboratories, Becton Dickinson, Sparks, MD, USA) plates and incubated at 35 ± 2 °C for 18 h. After incubation, a single colony was transferred to cation-adjusted Mueller-Hinton broth (CAMHB, Difco laboratories, Sparks, MD, USA) and incubated with shaking at 35 ± 2 °C for 18 h. To achieve the desired concentration, dilutions were performed with phosphate buffered saline (PBS; pH 7.4, Gibco, Grand Island, NY, USA) for colony-forming unit (CFU) assessment and CAMHB media for macro-dilution minimum inhibitory concentration (MIC) test, time-kill assay, synergy test, and protein synthesis inhibition test.

### 2.6. In Vitro Studies

#### 2.6.1. Macro-Dilution MIC Test

The MICs of SeNPs, SeNWs, and linezolid (LZD, Sigma-Aldrich, St. Louis, MO, USA) were determined by performing a two-fold macro-dilution broth method. First, 3.2 mg of SeNPs and SeNW were dissolved in 1 mL of PBS and then diluted to 8 concentrations from 3.2 to 0.025 mg/mL. Then, 3.2 mg of LZD was dissolved in 1 mL of PBS. It was diluted to 320 µg/mL and then diluted in 8 steps from 320 to 2.5 µg/mL. All strains that were grown on MHA plates at 35 ± 2 °C for 18 h were sub-cultured into 3 mL of CAMHB media at 35 ± 2 °C for 18 h. The cultured bacteria were diluted using CAMHB media to obtain bacterial cell densities of approximately 5 × 10^5^ CFU/mL. The bacterial concentration was determined by measuring the optical density at 600 nm with a spectrophotometer. The actual number of colonies was confirmed by diluting the bacterial culture solution that was diluted to the desired concentration to 10^3^ CFU/mL, dropping 100 µL on an MHA plate, spreading, and incubating. Nine hundred microliters of each diluted bacterial suspension was seeded in 5 mL polystyrene round bottom tubes (12 × 75 mm) containing 100 µL of serially diluted test compounds, which resulted in a 1/10 dilution of test compounds. As a positive control, 100 µL of PBS was added to 900 µL of bacterial solution, and 100 µL of PBS was added to 900 µL of CAMHB media as a negative control. Five-milliliter tubes were incubated with shaking at 35 ± 2 °C for 18 h. MICs were defined as the lowest concentrations that completely inhibited the growth of bacteria when viewed with the unaided eye. Since the MIC test was performed by finding the MIC values of the LZD for each bacterium several times, each MIC test including SeNPs and SeNWs was completed in one experiment.

#### 2.6.2. Time-Kill Assay

Time-kill studies were performed by the Clinical and Laboratory Standards Institute (CLSI) method [47]. All strains that were grown on MHA plates at 35 ± 2 °C for 18 h were sub-cultured into 3 mL of CAMHB media at 35 ± 2 °C for 18 h. The cultured bacteria were diluted using CAMHB media to obtain bacterial cell densities of approximately 5 × 10^5^ CFU/mL. The process of determining the concentration of bacteria was the same as that of the macro-dilution MIC test. A total of 1350 µL of each diluted bacterial suspension was seeded in 5 mL tubes containing 150 µL of SeNPs (concentration equivalent to 40×, 20×, 10× MIC against MSSA 6538 and MRSA 700699, and only 10× MIC against VRSA48). As a positive control, 150 µL of PBS was added to 1350 µL of bacterial suspension, and 150 µL of PBS was added to 1350 µL of CAMHB media as a negative control (vehicle). These 5 mL tubes were incubated with shaking at 35 ± 2 °C for 24 h. Aliquots (0.1 mL) of the cultures were removed at 0, 4, 8, and 24 h and then diluted with PBS to obtain the desired concentration. One hundred microliters of each diluted culture were spread on MHA plates. The plates were incubated at 35 ± 2 °C for 18 h, and then colonies were counted. In this experiment, the tendencies of results were confirmed through three repeated experiments, and the graph that best represents the trend among the three experiments was selected as a figure.

#### 2.6.3. Synergy Test of LZD and SeNPs

All strains that were grown on MHA plates at 35 ± 2 °C for 18 h were sub-cultured into 3 mL of CAMHB media at 35 ± 2 °C for 18 h. The cultured bacteria were diluted using CAMHB media to obtain bacterial cell densities of approximately 5 × 10^5^ CFU/mL. The process of assessing the concentration of bacteria was the same as that of the macro-dilution MIC test. A total of 1350 µL of each diluted bacterial suspension was seeded in 5 mL tubes containing 150 µL of SeNPs and LZD (their concentrations were equivalent to 10 times the MICs of each bacterium). For the Combi (SeNPs + LZD) group, 75 µL of SeNPs and LZD were added at a concentration of 20 times the MICs for each bacterium. As a positive control, 150 µL of PBS was added to 1350 µL of bacterial suspension, and 150 µL of PBS was added to 1350 µL of CAMHB media as a negative control (the negative control is not shown in the figure). These 5 mL tubes were incubated with shaking at 35 ± 2 °C for 24 h. Aliquots (0.1 mL) of the cultures were removed at 0 and 24 h and then diluted with PBS to obtain the desired concentration. One hundred microliters of each diluted culture was spread on MHA plates. The plates were incubated at 35 ± 2 °C for 18 h, and then colonies were counted. All experiments were conducted 3 times and averaged.

#### 2.6.4. Bacterial Protein Degradation Test

MRSA 700699 grown on MHA plates at 35 ± 2 °C for 18 h was sub-cultured into 3 mL of CAMHB media at 35 ± 2 °C for 18 h. The cultured bacteria were diluted using CAMHB media to obtain bacterial cell densities of approximately 5 × 10^5^ CFU/mL. The process of assessing the concentration of bacteria was the same as that of the macro-dilution MIC test. A total of 1350 µL of each diluted bacterial suspension was seeded in 5 mL tubes containing 150 µL of SeNPs and LZD (their concentrations were equivalent to 10 times the MICs for MRSA). For the Combi (SeNPs + LZD) group, 75 µL of SeNPs and LZD were added at a concentration of 20 times the MICs for MRSA. As a positive control, 150 µL of PBS was added to 1350 µL of bacterial suspension, and 150 µL of PBS was added to 1350 µL of CAMHB media as a negative control (the negative control is not shown in the figure). These 5 mL tubes were incubated with shaking at 35 ± 2 °C for 24 h. After incubation, 1 mL of each tube was transferred to 1.5 mL microtubes and centrifuged (1000× *g*, 30 min, 4 °C). The supernatant fluid was filtered through a 0.45 µm cellulose acetate filter (Avantor, Radnor, PA, USA). The filtrate was stored at −80 °C until LC-MS/MS analysis for calculation of fMLP. In this experiment, the tendencies of results were confirmed through three repeated experiments, and the graph that best represents the trend among the three experiments was selected as a figure.

### 2.7. Statistical Analysis

Synergy test data are shown as mean ± SD. In each experiment, n is the number of repeated trials. Statistical significance was determined using one-way ANOVA and Tukey’s multiple comparisons tests. GraphPad Prism 8 software (San Diego, CA, USA) was used.

## 3. Results and Discussion

### 3.1. Characterization of SeNPs and SeNWs

**Figure 1:**
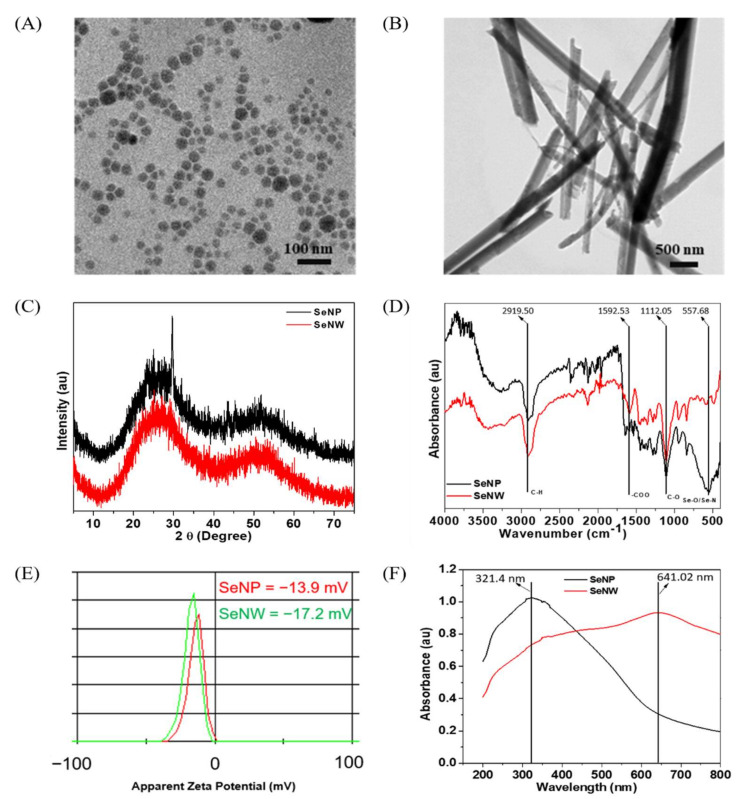
Characterizations of SeNPs and SeNWs. (**A**) TEM of SeNPs, (**B**) TEM image of SeNWs, (**C**) XRD patterns, showing the amorphous nature of sol-gel synthesized SeNPs and SeNWs, (**D**) FT-IR spectra of SeNPs and SeNWs, (**E**) ξ-potentials of SeNPs and SeNWs, (**F**) UV-visible spectra of SeNPs and SeNWs in DW.

The morphology and shape of the sol-gel synthesized SeNPs and SeNWs were observed by HR-TEM (Figure 1A,B). The SeNPs were spherical in shape with a size of 32.3 ± 5.6 nm, and SeNWs were 10–20 µm in size with an aspect ratio of 50–100. Next, we characterized the crystal phage structures of the synthesized selenium nanoparticles and nanowires. The XRD patterns of SeNPs and SeNWs are shown in Figure 1C and exhibit two broad peaks at 2θ = 20–30° and 45–55°, demonstrating a combination of crystalline and amorphous particles [48]. The FTIR spectra of the SeNPs and SeNWs showed four main peaks (Figure 1D). The sharp and intense peak at 2919.5 cm^−1^ corresponds to the –CH group, the peak at 1592.53 cm^−1^ corresponds to the –COO group, and the peaks at 1112.05 and 557.68 cm^−1^ correspond to –CO and Se-O groups, respectively [49,50]. Furthermore, zeta potential analysis confirmed that SeNPs and SeNWs have negative surface charges (Figure 1E). The surface charges of the SeNPs and SeNWs are −13.9 and −17.2 mV respectively, which is mainly due to the presence of -COO, -CO, and -OH chemical groups on the surface, as verified by FTIR analysis. The presence of the -COO, -CO, and -OH groups on the surface of the SeNPs/SeNWs can be understood based on the unreacted L-ascorbic acid and glucose. Ramamurthy et al. synthesized the selenium nanoparticles using the reduction method and found that C = C, NH2, COOH, CH2, and C = O chemical groups are responsible for the reduction of SeNPs [51]. Higher zeta potentials, either negative or positive, provide stable particle colloidal suspensions. Finally, the optical properties of the SeNPs and SeNWs were analyzed by UV-visible spectroscopy. The absorption spectra of the SeNPs and SeNWs are shown in Figure 1F. The SeNPs exhibit a broad absorption peak at 321.4 nm, and the SeNWs also exhibit broad peaks at 641.02 nm [52].

### 3.2. Broth Macro-Dilution MIC Test

**Figure 2:**
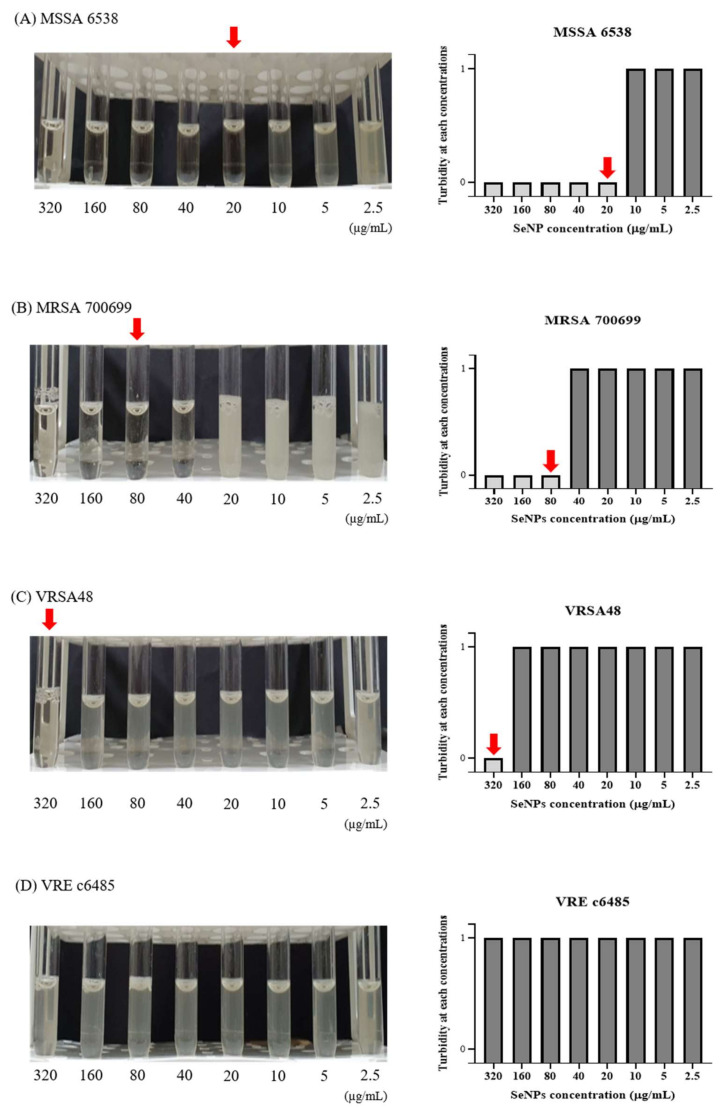
Macro-dilution MIC test for the SeNPs against MSSA, MRSA, VRSA, and VRE. (**A**) *S. aureus* ATCC 6538 (MSSA), (**B**) *S. aureus* ATCC 700699 (MRSA), (**C**) VRSA48, and (**D**) VRE c6485. In the 5 mL tube images, from left to right, the concentration of SeNPs was 320 to 2.5 µg/mL. The MICs were set to a concentration at which bacteria did not grow when viewed with the eye, as indicated by red arrows. In the bar graph, a turbidity value of 1 means that the bacteria have grown, and a turbidity value of 0 indicates that the bacteria have not grown when viewed with the eye. SeNPs showed MIC values of 20, 80, 320, and >320 μg/mL against MSSA, MRSA, VRSA, and VRE, respectively. Given that the concentration of nanoparticles cannot exceed 320 μg/mL, the experiments were conducted only for MSSA, MRSA, and VRSA from the subsequent experiment.

To investigate the antibacterial effects of SeNPs and SeNWs, broth macro-dilution MIC tests were performed against MSSA 6538, MRSA 700699, VRSA48, and VRE c6485 (Figure 2 and Supplementary Figure S1). The broth macro-dilution MIC test is a standard method described by CLSI [47]. The maximum concentration of SeNPs and SeNWs was set to 320 μg/mL. This setting was chosen because at a concentration of 640 μg/mL, the nanoparticles significantly stick to the tube wall during incubation, even if dispersion is performed by sonication. SeNPs showed MIC values of 20, 80, 320, and >320 μg/mL against MSSA 6538, MRSA 700699, VRSA48, and VRE c6485, respectively (Figure 2A–D). In the MIC test against MRSA700699, it looked clear even at a concentration of 40 μg/mL on the photograph, but it was slightly cloudy when viewed with the naked eye. Therefore, the concentration of 80 μg/mL was selected as the MIC. SeNWs showed MIC values of >320 μg/mL for all tested bacteria (Supplementary Figure S1). As mentioned above, since the experiment cannot be performed properly at a concentration of 640 μg/mL or greater, only the antibacterial activities of SeNPs against MSSA 6538, MRSA 700699, and VRSA48 were evaluated for subsequent experiments.

### 3.3. Time-Kill Assay and CFU Check

**Figure 3:**
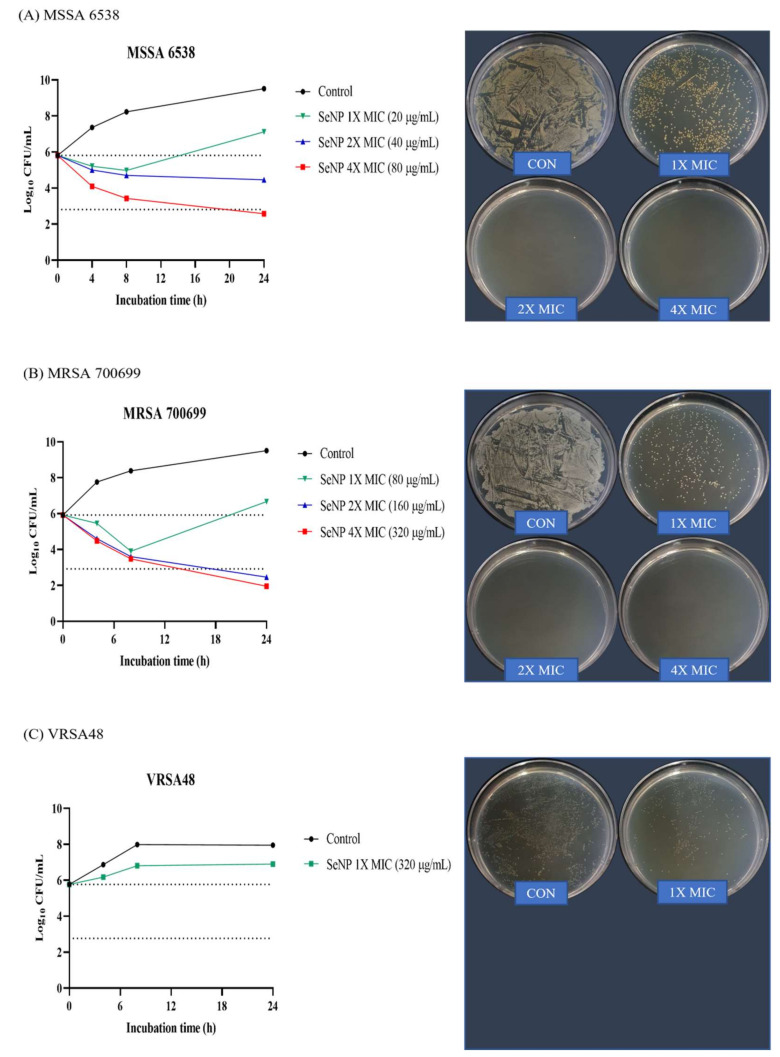
Time-kill assay and CFU check against MSSA, MRSA, and VRSA. (**A**) *S. aureus* ATCC 6538 (MSSA), (**B**) *S. aureus* ATCC 700699 (MRSA), and (**C**) VRSA48. Time-kill tests were performed at the concentrations of 1X, 2X, and 4X MIC values of SeNPs for each bacterium. The control was a bacterial solution without any antimicrobial agents. At 0, 4, 8, and 24 h, aliquots (100 µL) were taken from each group, diluted with PBS, and spread onto MHA plates with 100 µL of diluted solution to assess the CFU. Plate images were selected as plates capable of showing the first inoculation concentration of 10^5^ CFU/mL among spread plates. For MSSA, 4X MIC SeNPs showed bactericidal effects, and 2X MIC SeNPs showed bacteriostatic activity at 24 h. For MRSA, 2X and 4X MIC SeNPs showed bactericidal effects at 24 h. SeNPs at 1X MIC showed minimal antibacterial effects compared to the control against all tested bacteria at 24 h.

In vitro time-kill assays were performed against MSSA 6538, MRSA 700699, and VRSA48 to determine whether SeNPs had bactericidal or bacteriostatic activity. This method can be used to determine whether an antimicrobial agent is bactericidal or bacteriostatic. Bacterial reductions of 3 log of CFU/mL (99.9%) or greater compared to the initial inoculation concentration are considered bactericidal [53]. Alternatively, the tested compounds were considered bacteriostatic if the inoculum was reduced by 0–3 log of CFU/mL. For VRSA, only 10× MIC (3.2 mg/mL) SeNPs were prepared because SeNPs stick to the tube wall at high concentrations. Of the time points, 24 h, which shows the best difference between groups, was set as the time point for CFU images, and the dilution factor was set as the dilution factor capable of showing the initial inoculation concentration of 2.0~8.0 × 10^5^ CFU/mL. Therefore, if there were more bacteria than the initial inoculation concentration, a plate with a dense colony would be noted. Alternatively, if there were fewer bacteria, a cleaner plate would be evident. For MSSA 6538, 1× MIC SeNPs showed bacteriostatic effects up to 8 h, but the bacteria slightly increased at 24 h (Figure 3A). 2× MIC SeNPs showed bacteriostatic activity up to 24 h. SeNPs at 4× MIC showed bacteriostatic effects up to 8 h and bactericidal activity at 24 h. In CFU images, control and 1× MIC SeNPs showed relatively dense colonies, and 2× and 4× MIC SeNPs showed cleaner plates. For MRSA 700699, 1× MIC SeNPs showed bacteriostatic effects up to 8 h, but the bacteria slightly increased at 24 h (Figure 3B). SeNPs at 2× and 4× MIC showed bacteriostatic activity for up to 8 h and bactericidal effects at 24 h. In CFU images, similar to MSSA, control and 1× MIC SeNPs showed relatively dense colonies, and 2× and 4× MIC SeNPs showed clean plates. For VRSA48, 1X MIC SeNPs did not show bacteriostatic and bactericidal effects, and the number of bacteria were maintained at 10^6^ CFU/mL for 24 h (Figure 3C). However, it was considered an MIC because the bacteria did not grow when viewed with the naked eye. In CFU images, it was confirmed that 1× MIC SeNPs showed a lower number of colonies than the control.

### 3.4. Synergistic Effect of SeNPs and LZD

**Figure 4:**
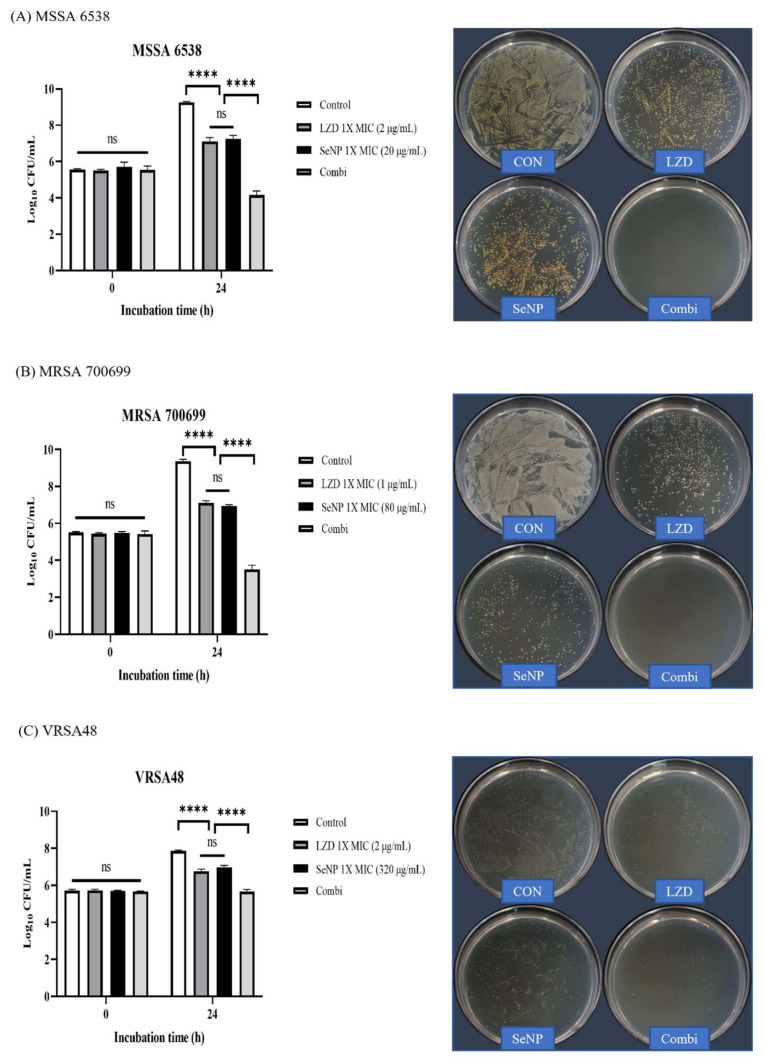
Synergy test and CFU assessment against MSSA, MRSA, and VRSA. (**A**) *S. aureus* ATCC 6538 (MSSA), (**B**) *S. aureus* ATCC 700699 (MRSA), and (**C**) VRSA48. The log values of CFU for each group are illustrated by the bar graphs as the mean ± SD (n = 3). The synergy tests were performed with 1× MIC values of LZD and SeNP for each bacterium. The control was a bacterial solution without any antimicrobial agents. The Combi group was mixed so that the concentrations of LZD and SeNPs were 1× MIC. The control was a bacterial solution without any antimicrobial agents. At 0 and 24 h, aliquots (100 µL) were taken from each group, diluted with PBS, and spread onto MHA plates with 100 µL of diluted solution to check the CFU. Plate images were selected as plates capable of showing the first inoculation concentration of 10^5^ CFU/mL among spread plates. For MSSA and MRSA, Combi showed a synergistic effect with a decrease of greater than 3 log values compared to the single most active drug. The Combi group yielded a clean plate. For VRSA, the combi group did not differ by more than 2 log values when compared to the single treated groups, indicating an additive effect rather than a synergistic effect. In the plate images, Combi yielded relatively clean plates compared to the group treated alone. *p*-values were calculated using one-way ANOVA with Tukey’s multiple comparisons test in this figure. ns, not significant; **** *p* < 0.0001.

One of the alternative strategies that deal with MDR bacterial infection is to treat conventional antibiotics with other types of antimicrobial agents. LZD is an oxazolidinone class of synthetic antibacterial agent that inhibits bacterial protein synthesis in the ribosome, and LZD is effective in treating MDR Gram-positive pathogens, such as MRSA, VRE, and VRSA [54,55,56,57]. Given that MRSA and VRSA were used in this study, LZD was selected among the conventional antibiotics. LZD showed synergistic effects with drugs targeting the bacterial membrane [58,59,60]. Since SeNPs can target the bacterial cellular membrane of *S. aureus*, we predicted that SeNPs and LZD would have a synergistic effect [61]. A synergistic effect was considered when the combination group had a reduction of 2 log or more compared to the most active single agent [62]. In the synergy test, given that it was difficult to see the synergistic effect at a concentration that was too good for antibacterial effects, the concentrations of SeNPs were set to 1× MIC, which did not show a bactericidal effect in the time-kill assay (Figure 3). The LZD concentrations were also set as a 1× MIC value. LZD MICs are presented in Supplementary Table S1. The time point for confirming the synergistic effect was 24 h, which showed a slight increase from the initial inoculation concentration in the time-kill assay (Figure 3). As noted in the time-kill assay, CFU image plates were spread with a dilution factor that could best show the initial inoculation concentration. The LZD and SeNP combination groups were referred to as the Combi group. Aliquots (0.1 mL) of each culture were removed at 0 and 24 h and then diluted with PBS to obtain the desired concentration. One hundred microliters of each diluted culture were spread on MHA plates. The plates were incubated at 35 ± 2 °C for 18 h, and then colonies were counted. The colony number is displayed as a log value on the left bar graph. For MSSA 6538 and MRSA 700699, the groups treated with LZD and SeNP respectively, showed some bacterial growth at 24 h (Figure 4A,B). However, the combination groups showed good synergistic effects by reducing the number of bacteria by a difference of 3 log or greater when compared to the most active single agent. In the CFU image, the combination group showed remarkably clean plates compared to the other groups. For VRSA48, the single treated groups showed a slight increase in the number of bacteria at 24 h, which is similar to that noted in the MSSA and MRSA groups (Figure 4C). The combination group significantly reduced CFU compared to the single treated groups, but it was considered an additive effect rather than a synergistic effect because there was no difference by more than 2 log. In the CFU image, the combination group showed a relatively small number of colonies compared to the other groups. In the next experiment, the amount of protein synthesis was analyzed to determine the mechanism through which the synergistic effect was produced.

### 3.5. Bacterial Protein Degradation Test

**Figure 5:**
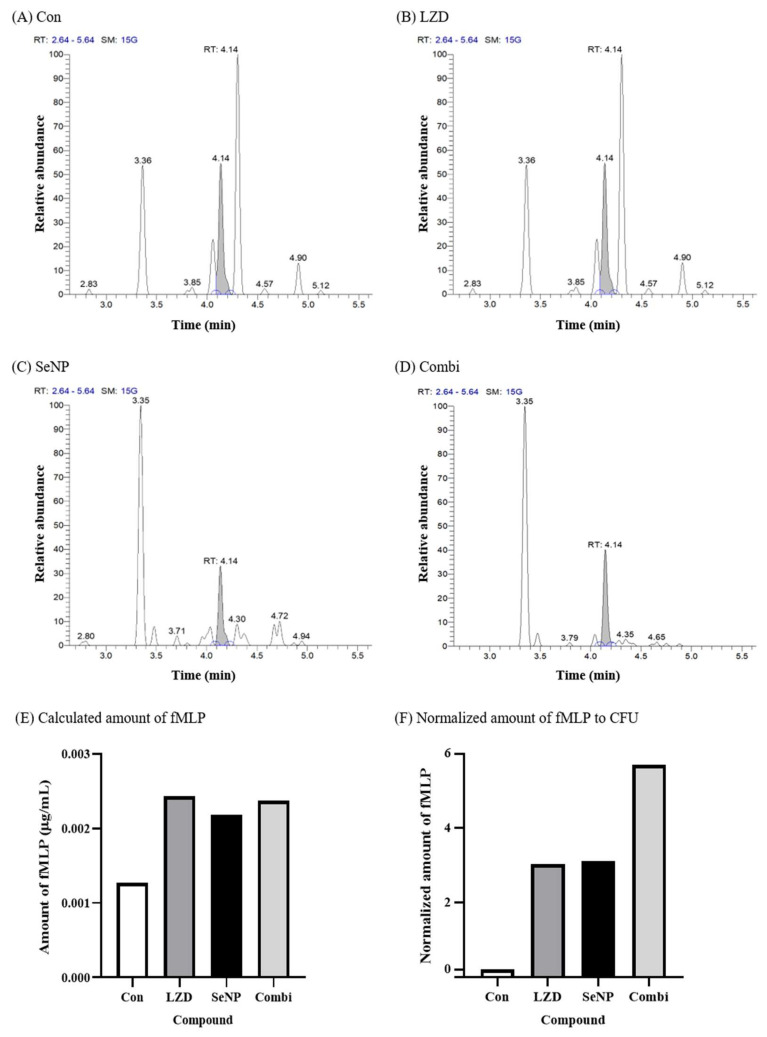
fMLP quantification by LC-MS/MS for the protein degradation test against MRSA 700699. (**A**) Control (without any antimicrobial agents), (**B**) LZD, (**C**) SeNP, (**D**) Combination (LZD + SeNP), (**E**) calculated amount of fMLP, and (**F**) normalized amount of fMLP to CFU. A protein degradation test was performed with only MRSA 700699, which showed the greatest synergistic effect in Figure 4. The CFU values used in (**B**) are shown in Figure 4B. The control was a bacterial solution without any antimicrobial agents. The concentrations of LZD and SeNP were the same as those used in Figure 4. In (**F**), as expected, the control group showed the lowest protein degradation value, and the combi group showed more protein degradation per bacterium than the single administration groups. Therefore, it seems that LZD and SeNPs showed synergistic effects through protein degradation.

A recent paradigm of drug discovery involves the identification of substances that can target proteins [63,64,65]. However, such studies on antibacterial agents are lacking. In this section, we assessed whether SeNPs exhibit antibacterial activity through protein degradation or contribute to protein degradation through synergistic effects with LZD. To confirm bacterial protein degradation, the amount of fMLP that was mainly induced by bacterial protein degradation was quantitatively analyzed by LC-MS/MS. This experiment was conducted with MRSA 700699, which showed the greatest synergistic effect in Figure 4. The culture process up to 24 h was the same as that noted for the synergy test. After 24 h of incubation, 1 mL of each tube was transferred to 1.5 mL microtubes and centrifuged (1000× *g*, 30 min, 4 °C). The supernatant fluid was filtered through a 0.45 µm cellulose acetate filter, and then the fMLP in the filtrate was quantitatively analyzed by LC-MS/MS. First, fMLP (≥97.0%, Sigma-Aldrich, USA) was analyzed as a standard material, and it was confirmed that fMLP was measured at 4.14 min. Therefore, the graph for each group was displayed for up to 5.64 min (Figure 5A–D). At 4.14 min, the fMLP peaks were filled in gray. In Figure 5A–D, given that the highest peak for each group was plotted as a value of 100 on the y-axis, fMLP amounts cannot be compared using the relative abundance or area of the graph. Each fMLP peak area before editing was compared to the area of standard material, and the amount of fMLP was calculated. Each calculated amount of fMLP is shown as a bar graph in Figure 5E. The fMLP values in the group containing antibiotics and SeNPs were increased approximately two-fold compared with that of the control group. However, the number of bacteria differed by greater than 10-fold compared to the control, and even the Combi group had a difference of greater than 10^5^ CFU at 24 h, as shown in Figure 4. Therefore, it was hypothesized that the amount of protein that was produced was different depending on the number of bacteria, and the amount of protein that was degraded was also affected. Hence, the calculated fMLP values were divided by the number of bacteria identified at 24 h, and all values were converted so that the control value was 1, and then expressed as a log value (Figure 5F). As expected, the control group showed the lowest protein degradation value, and the Combi group showed more protein degradation values than the single administration groups. Therefore, it seems that LZD and SeNPs showed synergistic effects through protein degradation. In addition, given that SeNP showed a similar degradation rate to that of LZD, it could likely to be used as a good antibacterial agent alone. Although we confirmed the antibacterial effects of SeNPs through macro-dilution MIC, time-kill, synergy, and bacterial protein degradation tests against MDR bacteria, some limitations still remain in this study. The first limitation is toxic issues of SeNPs. Se toxicity can occur through some mechanisms, such as the replacement of sulfur with selenium during protein synthesis and oxidative stress that can oxidize cellular thiols [40,41,66,67]. Although we consider SeNPs for topical uses such as dressings, additional studies are needed because this method may also cause toxicity problems. Second, although the antibacterial effects of SeNPs were confirmed through various tests, the number of bacterial strains used in this study was too small. In general, since antimicrobial substances can exhibit different antimicrobial effects depending on the bacterial strain, it is necessary to test with a larger number of strains to make the antibacterial effect of SeNPs more global [68,69,70]. Third, in addition to protein degradation, there may be factors affecting the synergistic effects of SeNPs and LZD. Further experiments are needed to better understand their synergistic effects, because nanoparticles exhibit antimicrobial effects through membrane rupture or disruption of intracellular components [26,27]. Additionally, studies of the mechanisms by which protein degradation occurs will be performed as the next step of this study. Finally, SeNPs do not dissolve well and have toxicity issues, so it is difficult to use it as a general injection method, such as intravenous injection. Therefore, we think that SeNPs have the potential as an antimicrobial agent in areas where it can be used locally, such as dressings. However, additional experiments are needed because toxicity can be a problem even with topical use. For these reasons, although we have seen that SeNPs can target MRSA and VRSA at concentrations above 320 µg/mL, it seems that finding the optimal concentration of SeNPs for an antibacterial effect in the clinical situation will require further study.

## 4. Conclusions

SeNPs exhibited antibacterial effects not only against MSSA but also against MDR bacteria, such as MRSA and VRSA. In addition, SeNPs showed a synergistic effect with LZD through protein degradation against MRSA. According to this experiment, both MRSA and VRSA can be targeted when the concentration of SeNPs is 320 µg/mL. However, due to limitations of this study such as toxicity of SeNPs and a small number of strains, the optimal concentration of SeNPs for an antibacterial effect could not be determined in this study. Therefore, to find the optimal SeNP concentration for an antibacterial effect, the number of strains needs to be increased, and further studies of SeNPs toxicity and synergistic mechanisms are needed. In conclusion, due to the toxicity and low solubility of SeNPs, topical use of SeNPs such as dressing rather than injections seems to have more potential in clinical settings as candidates for antibacterial substitutes and supplements against MDR bacteria.

## Data Availability

Not applicable.

## Supplementary Materials

The following are available online at https://www.mdpi.com/article/10.3390/biom11071028/s1, Figure S1: Macrodilution MIC test for the SeNWs against MSSA, MRSA, VRSA, and VRE. Table S1: Linezolid macrodilution MIC values against MSSA, MRSA, VRSA, and VRE.
