# Supplementary material for: Selenium Nanoparticles as Candidates for Antibacterial Substitutes and Supplements against Multidrug-Resistant Bacteria
Source: Biomolecules. 2021 Jul 14;11(7):1028. doi: 10.3390/biom11071028 (PMC8301847; doi:10.3390/biom11071028)
Supplement: Supplementary file 1 [file biomolecules-11-01028-s001.zip › biomolecules-1275176-supplementary.pdf]

Supplementary Data

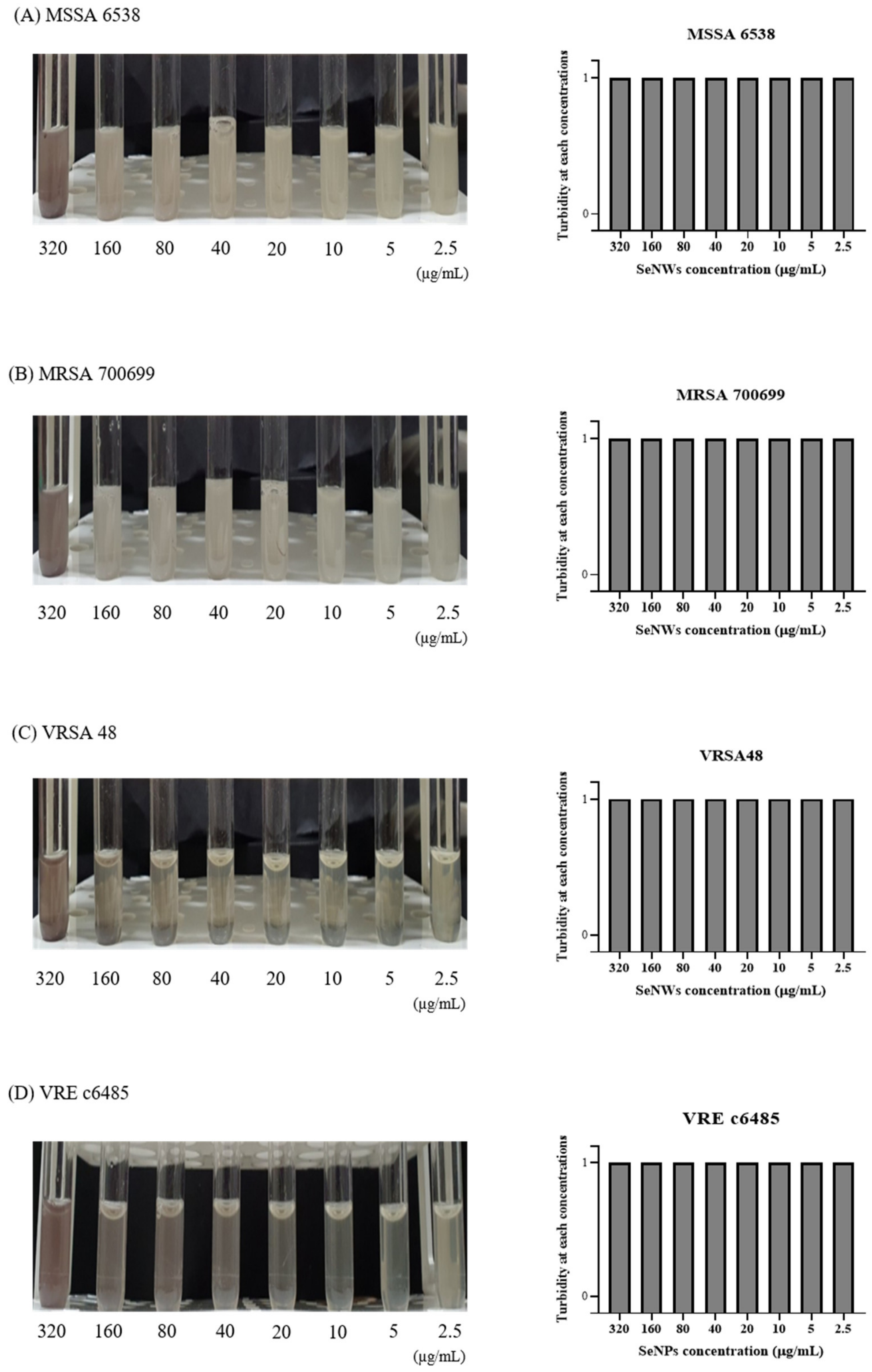

Figure S1. Macrodilution MIC test for the SeNWs against MSSA, MRSA, VRSA, and VRE. (**A**) *S. aureus* ATCC 6538 (MSSA), (**B**) *S. aureus* ATCC 700699 (MRSA), (**C**) VRSA48, and (**D**) VRE c6485. In the 5-mL tube images, the concentration of SeNPs was 320 to 2.5 μg/mL from left to right. In the bar graphs, 1 indicates that the medium is cloudy, and 0 means the medium is clean. SeNWs did not show antibacterial effects against all tested bacteria even at a concentration of 320 μg/mL. Therefore, SeNWs were excluded from the subsequent experiment.

Table S1. Linezolid macrodilution MIC values against MSSA, MRSA, VRSA, and VRE.

Strains	MIC (μg/mL)
S. aureus ATCC 6538 (MSSA)	2
S. aureus ATCC 700699 (MRSA)	1
VRSA48	2
E. faecalis c6485 (VRE)	1
